# (Di-2-pyridyl­amine-κ^2^ *N* ^2^,*N* ^2′^)diiodidopalladium(II)

**DOI:** 10.1107/S1600536812033946

**Published:** 2012-08-04

**Authors:** Kwang Ha

**Affiliations:** a School of Applied Chemical Engineering, The Research Institute of Catalysis, Chonnam National University, Gwangju 500-757, Republic of Korea

## Abstract

The Pd^II^ ion in the title complex, [PdI_2_(C_10_H_9_N_3_)], is four-coordinated in a distorted square-planar environment defined by the two pyridine N atoms of the chelating di-2-pyridyl­amine (dpa) ligand and two I^−^ anions. The dpa ligand is not planar, the dihedral angle between the pyridine rings being 51.2 (2)°. In the crystal, pairs of complex mol­ecules are assembled through inter­molecular N—H⋯I hydrogen bonds into dimeric species. The complexes are stacked in columns along the *b* axis and display several inter­molecular π–π inter­actions between the pyridine rings, with a shortest ring centroid–centroid distance of 3.957 (3) Å.

## Related literature

For the crystal structure of the related Pt^II^ complex [PtI_2_(dpa)], see: Ha (2012 ▶).
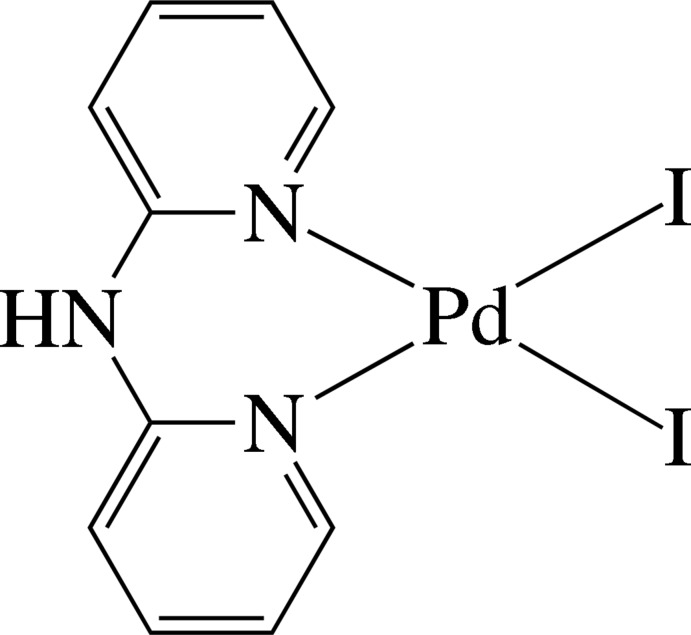


## Experimental

### 

#### Crystal data

[PdI_2_(C_10_H_9_N_3_)]
*M* *_r_* = 531.40
Monoclinic, 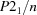

*a* = 8.2846 (8) Å
*b* = 9.7782 (9) Å
*c* = 16.5355 (14) Å
β = 102.344 (2)°
*V* = 1308.5 (2) Å^3^
*Z* = 4
Mo *K*α radiation
μ = 6.11 mm^−1^
*T* = 200 K
0.18 × 0.14 × 0.08 mm

#### Data collection

Bruker SMART 1000 CCD diffractometer
Absorption correction: multi-scan (*SADABS*; Bruker, 2000 ▶) *T*_min_ = 0.797, *T*_max_ = 1.000
9135 measured reflections
3152 independent reflections
2304 reflections with *I* > 2σ(*I*)
*R*_int_ = 0.032

#### Refinement

*R*[*F* ^2^ > 2σ(*F* ^2^)] = 0.028
*wR*(*F* ^2^) = 0.065
*S* = 1.01
3152 reflections
145 parameters
H-atom parameters constrained
Δρ_max_ = 1.17 e Å^−3^
Δρ_min_ = −0.78 e Å^−3^

### 

Data collection: *SMART* (Bruker, 2000 ▶); cell refinement: *SAINT* (Bruker, 2000 ▶); data reduction: *SAINT*; program(s) used to solve structure: *SHELXS97* (Sheldrick, 2008 ▶); program(s) used to refine structure: *SHELXL97* (Sheldrick, 2008 ▶); molecular graphics: *ORTEP-3* (Farrugia, 1997 ▶) and *PLATON* (Spek, 2009 ▶); software used to prepare material for publication: *SHELXL97*.

## Supplementary Material

Crystal structure: contains datablock(s) global, I. DOI: 10.1107/S1600536812033946/tk5137sup1.cif

Structure factors: contains datablock(s) I. DOI: 10.1107/S1600536812033946/tk5137Isup2.hkl

Additional supplementary materials:  crystallographic information; 3D view; checkCIF report

## Figures and Tables

**Table 1:** Selected bond lengths (Å)

Pd1—N1	2.068 (4)
Pd1—N3	2.067 (4)
Pd1—I1	2.5780 (5)
Pd1—I2	2.5957 (5)

**Table 2:** Hydrogen-bond geometry (Å, °)

*D*—H⋯*A*	*D*—H	H⋯*A*	*D*⋯*A*	*D*—H⋯*A*
N2—H2*N*⋯I2^i^	0.92	2.80	3.656 (4)	155

Symmetry code: (i) 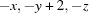.

**Fig. 1.**
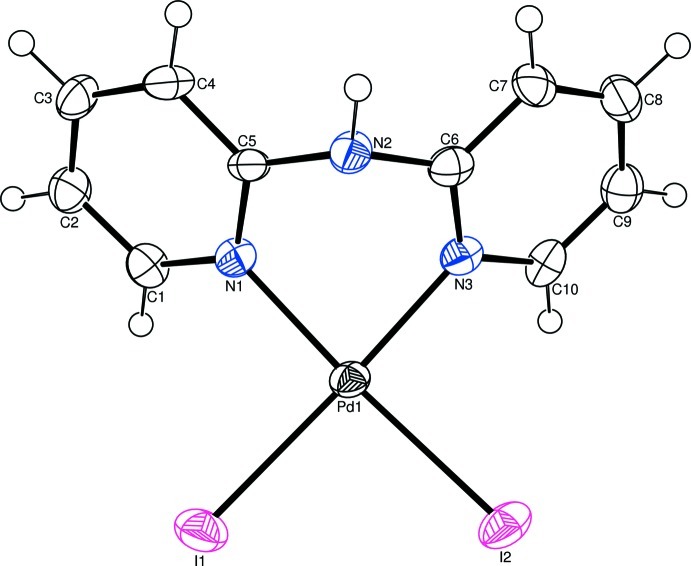
A structure detail of the title complex, with displacement ellipsoids drawn at the 50% probability level for non-H atoms.

**Fig. 2.**
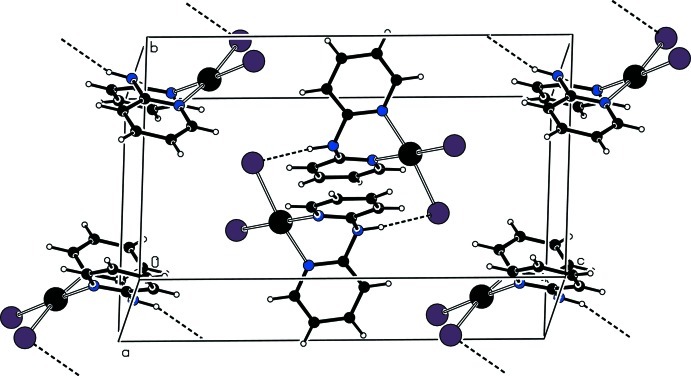
A view of the unit-cell contents of the title complex. Intermolecular N—H···I hydrogen-bond interactions are drawn with dashed lines.

## supplementary crystallographic information

### Comment

The title complex, [PdI_2_(dpa)] (dpa = di-2-pyridylamine, C_10_H_9_N_3_), is isomorphous with the previously reported Pt^II^ complex [PtI_2_(dpa)] (Ha, 2012).

The Pd^II^ ion is four-coordinated in a distorted square-planar environment defined by the two pyridine N atoms of the chelating dpa ligand and two I^-^ anions (Fig. 1). In the crystal, the dpa ligand is not planar. The dihedral angle between the least-squares planes of the pyridine rings is 51.2 (2)°. The nearly planar pyridine rings [maximum deviation = 0.031 (3) Å] are considerably inclined to the least-squares plane of the PdI_2_N_2_ unit [maximum deviation = 0.081 (1) Å], making dihedral angles of 46.5 (1)° and 51.6 (1)°. The Pd—N and Pd—I bond lengths are nearly equivalent, respectively (Table 1). Pairs of complex molecules are assembled through intermolecular N—H···I hydrogen bonds into dimeric species. (Fig. 2 and Table 2). The complexes are stacked in columns along the *b* axis and display several intermolecular π-π interactions between the pyridine rings, with a shortest ring centroid-centroid distance of 3.957 (3) Å.

### Experimental

To a solution of Na_2_PdCl_4_ (0.1461 g, 0.497 mmol) and KI (0.7811 g, 4.705 mmol) in MeOH (30 ml) was added di-2-pyridylamine (0.0862 g, 0.519 mmol) followed by stirring at room temperature for 5 h. The formed precipitate was separated by filtration and washed with H_2_O and acetone, and dried at 50 °C, to give a dark-orange powder (0.2322 g). Crystals suitable for X-ray analysis were obtained by slow evaporation from a CH_3_NO_2_ solution held at room temperature.

### Refinement

Carbon-bound H atoms were positioned geometrically and allowed to ride on their respective parent atoms: C—H = 0.95 Å and *U*_iso_(H) = 1.2*U*_eq_(C). The nitrogen-bound H atom was located from a Fourier difference map and then allowed to ride on its parent atom in the final cycles of refinement with N—H = 0.92 Å and *U*_iso_(H) = 1.5*U*_eq_(N). The highest peak (1.17 e Å^-3^) and the deepest hole (-0.78 e Å^-3^) in the difference Fourier map are located 1.99 Å and 0.82 Å, respectively, from the atoms H4 and I2. A number of reflections were omitted from the final cycles of refinement owing to poor agreement.

### Crystal data

**Table:**

[PdI_2_(C_10_H_9_N_3_)]	*F*(000) = 968
*M**_r_* = 531.40	*D*_x_ = 2.697 Mg m^−^^3^
Monoclinic, *P*2_1_/*n*	Mo *K*α radiation, λ = 0.71073 Å
Hall symbol: -P 2yn	Cell parameters from 4274 reflections
*a* = 8.2846 (8) Å	θ = 2.4–28.3°
*b* = 9.7782 (9) Å	µ = 6.11 mm^−^^1^
*c* = 16.5355 (14) Å	*T* = 200 K
β = 102.344 (2)°	Block, red
*V* = 1308.5 (2) Å^3^	0.18 × 0.14 × 0.08 mm
*Z* = 4	

### Data collection

**Table:**

Bruker SMART 1000 CCD diffractometer	3152 independent reflections
Radiation source: fine-focus sealed tube	2304 reflections with *I* > 2σ(*I*)
Graphite monochromator	*R*_int_ = 0.032
φ and ω scans	θ_max_ = 28.3°, θ_min_ = 2.4°
Absorption correction: multi-scan (*SADABS*; Bruker, 2000)	*h* = −10→11
*T*_min_ = 0.797, *T*_max_ = 1.000	*k* = −13→11
9135 measured reflections	*l* = −22→18

### Refinement

**Table:**

Refinement on *F*^2^	Primary atom site location: structure-invariant direct methods
Least-squares matrix: full	Secondary atom site location: difference Fourier map
*R*[*F*^2^ > 2σ(*F*^2^)] = 0.028	Hydrogen site location: inferred from neighbouring sites
*wR*(*F*^2^) = 0.065	H-atom parameters constrained
*S* = 1.01	*w* = 1/[σ^2^(*F*_o_^2^) + (0.0239*P*)^2^] where *P* = (*F*_o_^2^ + 2*F*_c_^2^)/3
3152 reflections	(Δ/σ)_max_ = 0.001
145 parameters	Δρ_max_ = 1.17 e Å^−^^3^
0 restraints	Δρ_min_ = −0.78 e Å^−^^3^

### Special details

**Table:**

Geometry. All e.s.d.'s (except the e.s.d. in the dihedral angle between two l.s. planes) are estimated using the full covariance matrix. The cell e.s.d.'s are taken into account individually in the estimation of e.s.d.'s in distances, angles and torsion angles; correlations between e.s.d.'s in cell parameters are only used when they are defined by crystal symmetry. An approximate (isotropic) treatment of cell e.s.d.'s is used for estimating e.s.d.'s involving l.s. planes.
Refinement. Refinement of *F*^2^ against ALL reflections. The weighted *R*-factor *wR* and goodness of fit *S* are based on *F*^2^, conventional *R*-factors *R* are based on *F*, with *F* set to zero for negative *F*^2^. The threshold expression of *F*^2^ > σ(*F*^2^) is used only for calculating *R*-factors(gt) *etc*. and is not relevant to the choice of reflections for refinement. *R*-factors based on *F*^2^ are statistically about twice as large as those based on *F*, and *R*- factors based on ALL data will be even larger.

### Fractional atomic coordinates and isotropic or equivalent isotropic displacement parameters (Å^2^)

**Table:**

	*x*	*y*	*z*	*U*_iso_*/*U*_eq_	
Pd1	0.07287 (4)	0.84262 (4)	0.15317 (2)	0.02380 (10)	
I1	−0.13829 (4)	0.86318 (4)	0.24664 (2)	0.03867 (11)	
I2	0.24276 (4)	1.04417 (4)	0.230708 (19)	0.03719 (11)	
N1	−0.0699 (5)	0.6997 (4)	0.0777 (2)	0.0248 (9)	
N2	−0.0086 (5)	0.8273 (4)	−0.0325 (2)	0.0273 (9)	
H2N	−0.0376	0.8473	−0.0881	0.041*	
N3	0.2299 (5)	0.8278 (4)	0.0718 (2)	0.0269 (9)	
C1	−0.1482 (6)	0.5937 (5)	0.1053 (3)	0.0288 (11)	
H1	−0.1266	0.5751	0.1630	0.035*	
C2	−0.2578 (6)	0.5121 (5)	0.0525 (3)	0.0340 (12)	
H2	−0.3109	0.4381	0.0735	0.041*	
C3	−0.2900 (6)	0.5389 (5)	−0.0318 (3)	0.0336 (12)	
H3	−0.3679	0.4853	−0.0692	0.040*	
C4	−0.2078 (6)	0.6437 (5)	−0.0604 (3)	0.0299 (11)	
H4	−0.2266	0.6625	−0.1180	0.036*	
C5	−0.0973 (5)	0.7219 (5)	−0.0047 (3)	0.0227 (10)	
C6	0.1623 (6)	0.8357 (5)	−0.0085 (3)	0.0267 (11)	
C7	0.2600 (6)	0.8499 (5)	−0.0670 (3)	0.0301 (12)	
H7	0.2097	0.8615	−0.1238	0.036*	
C8	0.4280 (6)	0.8469 (5)	−0.0424 (3)	0.0352 (13)	
H8	0.4961	0.8574	−0.0815	0.042*	
C9	0.4975 (6)	0.8284 (5)	0.0412 (3)	0.0343 (12)	
H9	0.6138	0.8203	0.0597	0.041*	
C10	0.3965 (6)	0.8219 (5)	0.0962 (3)	0.0293 (11)	
H10	0.4446	0.8130	0.1535	0.035*	

### Atomic displacement parameters (Å^2^)

**Table:**

	*U*^11^	*U*^22^	*U*^33^	*U*^12^	*U*^13^	*U*^23^
Pd1	0.0271 (2)	0.0261 (2)	0.01677 (18)	−0.00202 (16)	0.00160 (15)	0.00029 (15)
I1	0.0427 (2)	0.0497 (3)	0.02587 (19)	0.00181 (17)	0.01239 (16)	−0.00231 (16)
I2	0.0485 (2)	0.0335 (2)	0.02414 (18)	−0.00877 (16)	−0.00439 (15)	−0.00119 (14)
N1	0.027 (2)	0.023 (2)	0.023 (2)	0.0005 (17)	0.0030 (17)	0.0009 (17)
N2	0.030 (2)	0.031 (3)	0.018 (2)	−0.0062 (18)	0.0007 (17)	0.0053 (17)
N3	0.030 (2)	0.026 (2)	0.023 (2)	−0.0013 (18)	0.0020 (18)	−0.0016 (17)
C1	0.032 (3)	0.024 (3)	0.032 (3)	0.002 (2)	0.010 (2)	0.005 (2)
C2	0.036 (3)	0.028 (3)	0.039 (3)	−0.007 (2)	0.011 (2)	−0.002 (2)
C3	0.032 (3)	0.029 (3)	0.039 (3)	−0.009 (2)	0.006 (2)	−0.011 (2)
C4	0.042 (3)	0.028 (3)	0.019 (2)	−0.001 (2)	0.006 (2)	−0.004 (2)
C5	0.024 (2)	0.026 (3)	0.018 (2)	0.002 (2)	0.0038 (19)	−0.0044 (19)
C6	0.036 (3)	0.019 (3)	0.025 (3)	0.000 (2)	0.005 (2)	0.000 (2)
C7	0.043 (3)	0.023 (3)	0.028 (3)	−0.004 (2)	0.015 (2)	0.003 (2)
C8	0.040 (3)	0.029 (3)	0.043 (3)	−0.004 (2)	0.022 (3)	−0.003 (2)
C9	0.031 (3)	0.029 (3)	0.043 (3)	0.000 (2)	0.008 (2)	0.001 (2)
C10	0.024 (2)	0.022 (3)	0.038 (3)	0.000 (2)	−0.001 (2)	−0.001 (2)

### Geometric parameters (Å, º)

**Table:**

Pd1—N1	2.068 (4)	C2—H2	0.9500
Pd1—N3	2.067 (4)	C3—C4	1.370 (7)
Pd1—I1	2.5780 (5)	C3—H3	0.9500
Pd1—I2	2.5957 (5)	C4—C5	1.382 (6)
N1—C5	1.351 (5)	C4—H4	0.9500
N1—C1	1.353 (6)	C6—C7	1.394 (6)
N2—C6	1.389 (6)	C7—C8	1.365 (7)
N2—C5	1.399 (6)	C7—H7	0.9500
N2—H2N	0.9200	C8—C9	1.390 (7)
N3—C6	1.329 (6)	C8—H8	0.9500
N3—C10	1.354 (6)	C9—C10	1.362 (7)
C1—C2	1.372 (7)	C9—H9	0.9500
C1—H1	0.9500	C10—H10	0.9500
C2—C3	1.388 (7)		
			
N3—Pd1—N1	85.31 (15)	C2—C3—H3	120.5
N3—Pd1—I1	176.37 (11)	C3—C4—C5	119.4 (4)
N1—Pd1—I1	92.33 (10)	C3—C4—H4	120.3
N3—Pd1—I2	91.40 (11)	C5—C4—H4	120.3
N1—Pd1—I2	172.21 (11)	N1—C5—C4	121.9 (4)
I1—Pd1—I2	90.598 (16)	N1—C5—N2	117.5 (4)
C5—N1—C1	118.3 (4)	C4—C5—N2	120.6 (4)
C5—N1—Pd1	116.8 (3)	N3—C6—N2	117.9 (4)
C1—N1—Pd1	124.6 (3)	N3—C6—C7	121.1 (4)
C6—N2—C5	121.6 (4)	N2—C6—C7	121.0 (4)
C6—N2—H2N	107.8	C8—C7—C6	119.8 (5)
C5—N2—H2N	116.2	C8—C7—H7	120.1
C6—N3—C10	119.0 (4)	C6—C7—H7	120.1
C6—N3—Pd1	117.2 (3)	C7—C8—C9	118.6 (5)
C10—N3—Pd1	123.6 (3)	C7—C8—H8	120.7
N1—C1—C2	122.1 (4)	C9—C8—H8	120.7
N1—C1—H1	118.9	C10—C9—C8	119.1 (5)
C2—C1—H1	118.9	C10—C9—H9	120.4
C1—C2—C3	119.2 (5)	C8—C9—H9	120.4
C1—C2—H2	120.4	N3—C10—C9	122.1 (5)
C3—C2—H2	120.4	N3—C10—H10	118.9
C4—C3—C2	119.1 (5)	C9—C10—H10	118.9
C4—C3—H3	120.5		
			
N3—Pd1—N1—C5	−46.2 (3)	C3—C4—C5—N1	−1.3 (7)
I1—Pd1—N1—C5	131.0 (3)	C3—C4—C5—N2	178.3 (4)
N3—Pd1—N1—C1	139.7 (4)	C6—N2—C5—N1	52.2 (6)
I1—Pd1—N1—C1	−43.0 (4)	C6—N2—C5—C4	−127.4 (5)
N1—Pd1—N3—C6	48.2 (4)	C10—N3—C6—N2	173.5 (4)
I2—Pd1—N3—C6	−124.7 (3)	Pd1—N3—C6—N2	−11.9 (6)
N1—Pd1—N3—C10	−137.4 (4)	C10—N3—C6—C7	−5.6 (7)
I2—Pd1—N3—C10	49.7 (4)	Pd1—N3—C6—C7	169.1 (4)
C5—N1—C1—C2	−2.3 (7)	C5—N2—C6—N3	−50.6 (6)
Pd1—N1—C1—C2	171.7 (4)	C5—N2—C6—C7	128.5 (5)
N1—C1—C2—C3	−0.1 (8)	N3—C6—C7—C8	4.2 (7)
C1—C2—C3—C4	1.9 (8)	N2—C6—C7—C8	−174.8 (5)
C2—C3—C4—C5	−1.2 (8)	C6—C7—C8—C9	0.6 (8)
C1—N1—C5—C4	3.0 (7)	C7—C8—C9—C10	−3.9 (8)
Pd1—N1—C5—C4	−171.4 (4)	C6—N3—C10—C9	2.2 (7)
C1—N1—C5—N2	−176.6 (4)	Pd1—N3—C10—C9	−172.1 (4)
Pd1—N1—C5—N2	9.0 (5)	C8—C9—C10—N3	2.6 (8)

### Hydrogen-bond geometry (Å, º)

**Table:**

*D*—H···*A*	*D*—H	H···*A*	*D*···*A*	*D*—H···*A*
N2—H2*N*···I2^i^	0.92	2.80	3.656 (4)	155

Symmetry code: (i) −*x*, −*y*+2, −*z*.
